# Supplementary material for: Development of a computer-aided design software for the quantitative evaluation of aesthetic damage
Source: PLoS One. 2019 Dec 18;14(12):e0226322. doi: 10.1371/journal.pone.0226322 (PMC6919621; doi:10.1371/journal.pone.0226322)
Supplement: S1 Code site — 1. (ZIP) [file pone.0226322.s001.zip › [EESC_jr][Projeto]Relatorio_07_21/reports/templates_zip/gentelella-master/vendors/google-code-prettify/styles/demo.html]

```
<script type="text/javascript">
// Say hello world until the user starts questioning
// the meaningfulness of their existence.
function helloWorld(world) {
  for (var i = 42; --i >= 0;) {
    alert('Hello ' + String(world));
  }
}
</script>
<style>
p { color: pink }
b { color: blue }
u { color: "umber" }
</style>
```
